# Elevated Asporin expression in human atherosclerotic plaques promotes their stability and reduces the risk for cardiovascular events

**DOI:** 10.1093/cvr/cvag015

**Published:** 2026-01-20

**Authors:** Panagiotis Fountas, Chrysostomi Gialeli, Nicoline W Thorsen, Dianne Acoba, Jiangming Sun, Luke F Gamon, Annelie Shami, Mihaela Nitulescu, Ana Persson, Eva Bengtsson, Michael J Davies, Andreas Edsfeldt, Claudia Goettsch, Isabel Gonçalves

**Affiliations:** Cardiovascular Research Translational Studies, Department of Clinical Sciences Malmö, Lund University, Malmö, Sweden; Cardiovascular Research Translational Studies, Department of Clinical Sciences Malmö, Lund University, Malmö, Sweden; Cardiovascular Research Translational Studies, Department of Clinical Sciences Malmö, Lund University, Malmö, Sweden; Department of Biomedical Sciences, University of Copenhagen, Copenhagen, Denmark; Cardiovascular Research Translational Studies, Department of Clinical Sciences Malmö, Lund University, Malmö, Sweden; Cardiovascular Research Translational Studies, Department of Clinical Sciences Malmö, Lund University, Malmö, Sweden; Cardiovascular Research Translational Studies, Department of Clinical Sciences Malmö, Lund University, Malmö, Sweden; Department of Biomedical Sciences, University of Copenhagen, Copenhagen, Denmark; Cardiovascular Research Translational Studies, Department of Clinical Sciences Malmö, Lund University, Malmö, Sweden; Cardiovascular Research Translational Studies, Department of Clinical Sciences Malmö, Lund University, Malmö, Sweden; Cardiovascular Research Translational Studies, Department of Clinical Sciences Malmö, Lund University, Malmö, Sweden; Cardiovascular Research Translational Studies, Department of Clinical Sciences Malmö, Lund University, Malmö, Sweden; Department of Biomedical Science, Faculty of Health and Society, Malmö University, Malmö, Sweden; Biofilms–Research Center for Biointerfaces, Malmö University, Malmö, Sweden; Department of Biomedical Sciences, University of Copenhagen, Copenhagen, Denmark; Cardiovascular Research Translational Studies, Department of Clinical Sciences Malmö, Lund University, Malmö, Sweden; Department of Cardiology, Skåne University Hospital, Malmö, Sweden; Wallenberg Center for Molecular Medicine, Lund University, Malmö, Sweden; Department of Internal Medicine I, Division of Cardiology, Medical Faculty, RWTH Aachen University, Germany; Faculty of Medicine Carl Gustav Carus, Institute of Physiology, Technical University Dresden, Dresden, Germany; Cardiovascular Research Translational Studies, Department of Clinical Sciences Malmö, Lund University, Malmö, Sweden; Department of Cardiology, Skåne University Hospital, Malmö, Sweden

**Keywords:** Asporin, Human atherosclerosis, Plaque, Calcification

## Abstract

**Aims:**

Vascular atherosclerotic calcification is a pathological process marked by the abnormal deposition of calcium minerals in the intima. Asporin (ASPN) is a small leucine-rich proteoglycan which interacts with collagen and calcium. Due to its role in matrix mineralization, we hypothesized that ASPN might act as a regulator of vascular calcification, thereby promoting atherosclerotic plaque stability.

**Methods and results:**

ASPN protein, analysed by ELISA, was quantified in 176 carotid endarterectomy plaques (Carotid Plaque Imaging Project cohort, including 98 patients with cerebrovascular symptoms and 78 asymptomatic patients). Plaque composition was assessed by histological, biochemical, and immunological assays, along with bulk RNA sequencing, to investigate the role of ASPN in atherosclerosis. Patients donating plaques were followed up for post-operative cardiovascular events, median follow-up 6.58 years. The effect of ASPN on smooth muscle cell (SMC) differentiation and matrix mineralization was investigated *in vitro* using human vascular SMCs overexpressing ASPN. Increased ASPN protein levels were observed in plaques from asymptomatic patients compared with patients with cerebrovascular symptoms. ASPN protein levels were positively associated with markers of plaque stability and regulation of extracellular matrix remodelling while showing an inverse relationship with calcification. Patients with high intraplaque ASPN had a lower risk for future cardiovascular events. Mechanistically, ASPN overexpression in vascular SMCs reduced matrix mineralization *in vitro*, supporting its potential role in plaque stabilization.

**Conclusion:**

ASPN is a regulator of vascular calcification in atherosclerosis, promoting a plaque phenotype that is less prone to rupture. Additionally, high ASPN levels are associated with fewer future cardiovascular events.

## Graphical Abstract

**Graphical Abstract:**
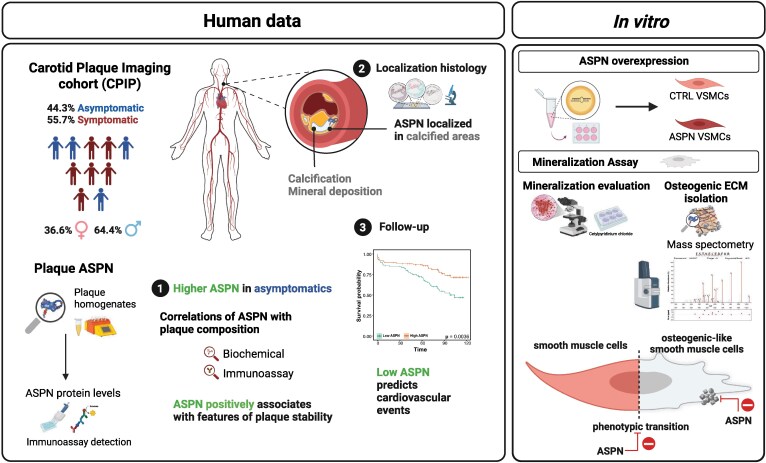
High ASPN levels in atherosclerotic plaques are associated with reduced vascular calcification and a more stable plaque phenotype, while low ASPN levels predict future cardiovascular events. Created in BioRender. Gialeli, C. (2025). https://BioRender.com/j15d480

**Time of primary review: 43 days**

## 1. Introduction

Atherosclerosis is a chronic inflammatory process leading to the formation of plaques in the intima of the arteries.^1^ Plaque formation involves lipid accumulation creating a necrotic core, active inflammation, cell death, calcification, and extensive extracellular matrix (ECM) remodelling with the formation of a fibrous cap. Destabilizing features, such as a large lipid-rich necrotic core, covered by a thin inflamed cap, contribute to an increased risk of plaque rupture and subsequent acute cardiovascular complications.^2,3^ Vascular calcification is characterized by an inappropriate and pathological deposition of calcium minerals within the arterial wall.^4^ Mineral deposition can occur in different locations of the vasculature, but in the intima it is closely linked to atherosclerosis, hyperlipidaemia, and chronic inflammation.^5^ Smooth muscle cells (SMCs) are among the cells that orchestrate vascular calcification via their osteogenic transition and production of an ECM scaffold for mineral deposition.^6,7^

Asporin (ASPN), also known as periodontal ligament-associated protein 1 (PLAP1), is a small leucine-rich proteoglycan (SLRP).^8^ It is expressed in various tissues, with higher levels found in articular cartilage, aorta, uterus, heart, and liver.^9^ In these tissues, ASPN expression is cell type-specific, with chondrocytes, SMCs, and fibroblasts as the main sources, while other cell types contribute less and in a context-specific manner.^9,10^ Unlike the other members of class I SLRPs, ASPN lacks attached glycosaminoglycan chains but features in its N-terminal domain multiple conserved aspartate (D)-repeats, capable of binding calcium.^11,12^ D-rich regions of ASPN bind to type II collagen, while the leucine-rich repeat (LRR) 10–12 binds to type I collagen and regulates collagen fibril formation.^11^ ASPN interacts with transforming growth factor-β (TGF-β)^13,14^ and BMP2,^15,16^ regulating their activity.^17^ Previous studies have shown that ASPN inhibits osteoblastic differentiation and matrix mineralization in periodontal ligament cells.^16,18^ Interestingly, ASPN has been detected in proteomic analyses of atherosclerotic plaques from carotid arteries,^19^ and reported to be associated with ‘hard’ plaques together with other mineralization related proteins.^20^

As ASPN plays a role in ECM mineralization, we hypothesized that ASPN would function as a negative regulator of vascular calcification and promote plaque stability. Besides *in vitro* experiments, in the present study, we quantified ASPN in plaques from patients undergoing carotid endarterectomy and examined their associations with plaque features, calcification deposits, and the possibility of predicting future post-operative cardiovascular events prospectively.

## 2. Methods

### 2.1 Carotid plaque imaging project biobank

Carotid plaques (*n* = 176) were collected from patients included in the Carotid Plaque Imaging Project (CPIP) cohort who underwent carotid endarterectomy at the Vascular Department of Skåne University Hospital (Malmö, Sweden) between 2005 and 2012. Symptomatic patients had carotid artery stenosis >70% and suffered from *amaurosis fugax*, transient ischaemic attack, or ischaemic stroke, whereas asymptomatic patients had >80% stenosis without symptoms within 6 months prior to surgery. Data on cardiovascular risk factors and medication were collected, including hypertension (systolic blood pressure >140 mmHg), diabetes, current smoking status, and plasma levels of total cholesterol, high-density lipoprotein, low-density lipoprotein, and triglycerides. Ethical approval was granted by the Swedish Ethical Review Authority (472/2005; 2014/904; 60/2008; 2023-05910-01). The study was carried out in accordance with the principles of the Declaration of Helsinki (1975). All study participants provided written informed consent. The sharing of study datasets containing pseudonymized participant data is subject to restrictions outlined in the ethical permit and in accordance with general data protection regulations. [GDPR (EU) 2016/679]. The clinical characteristics of the patients included for plaque ASPN measurements are presented in *Table 1*. A study design flow chart is presented in Supplementary material online, *Figure S1*.

**Table 1:** Baseline clinical characteristics in relation to ASPN levels

	Low ASPN (*n* = 88)	High ASPN (*n* = 88)	*P*
Age, years (SD)	71.36 (± 8.23)	68.90 (± 8.13)	0.04
Sex: men, *n* (%)	69 (78.4%)	50 (56.8%)	0.004
Degree of stenosis (IQR)	90.00 (80.00–95.00)	90.00 (80.00–95.00)	0.56
Symptoms, *n* (%)	58 (65.9%)	40 (45.5%)	0.01
Diabetes, *n* (%)	39 (44.3%)	23 (26.1%)	0.02
Hypertension, *n* (%)	68 (77.3%)	67 (76.1%)	1
Current smoking, *n* (%)	22 (25.0%)	34 (38.6%)	0.08
Body Mass Index (IQR)	26.50 (24.00–30.00)	26.35 (23.95–28.75)	0.699
hsCRP (mg/L) (IQR)	3.00 (0.85–5.85)	3.00 (1.40–5.00)	0.966
Cholesterol (mmol/L) (IQR)	4.25 (3.50–5.00)	4.30 (3.60–5.10)	0.568
Triglycerides (mmol/L) (IQR)	1.40 (1.00–1.70)	1.30 (1.00–1.80)	0.98
HDL (mmol/L) (IQR)	1.09 (0.88–1.30)	1.06 (0.88–1.40)	0.908
LDL (mmol/L) (IQR)	2.50 (2.00–3.06)	2.50 (1.81–3.38)	0.886
Statin treatment, *n* (%)	78 (88.6%)	78 (88.6%)	1

Mean with standard deviation (SD) for normally distributed continuous variables, median with interquartile range (IQR) for non-normally distributed continuous variables and frequencies (percentages) for categorical variables are presented. Characteristics of participants are compared using *t*-test for continuous normally distributed variables, Mann–Whitney *U* test for continuous non-normally distributed variables and Pearson Chi-squared tests for categorical variables.

HDL, high-density lipoproteins; LDL, low-density lipoproteins; hsCRP, high-sensitivity C-reactive protein.

### 2.2 Plaque homogenate analysis

Plaque homogenates of 176 patients were prepared as described previously.^21^ Intra-plaque measurements were standardized to wet plaque weight. TGF-β isoforms β1, β2, and β3 were measured with the Milliplex Map TGF-β Magnetic Bead 3 Plex Kit—Immunology Multiplex Assay TGFBMAG-64K-03 (Merck Millipore, Darmstadt, Germany) as described previously.^22^ Profiling of matrix turnover regulators, including matrix metalloproteinases (MMPs) -2, -3, -9, -10 and tissue inhibitor of matrix metalloproteinases (TIMPs) -1, -2, and -3 were measured using the Mesoscale human MMP ultra-sensitive kit (Meso Scale Diagnostics, Rockville, MD, USA) and Milliplex Map Human TIMP Magnetic Bead Panel HTMP2MAG-54K (Merck Millipore), respectively, as described previously.^23^ Collagen and elastin levels were measured using the Sircol Soluble Collagen Assay (Biocolor Ltd, Belfast, United Kingdom) and the Fastin Elastin Assay (Biocolor Ltd), respectively, following the manufacturer’s protocols.^24^ ASPN was detected in plaque homogenates using a Human Asporin ELISA Kit (Novus Biologicals part of Bio-Techne Ltd., Abingdon, United Kingdom), according to the manufacturer’s protocol.

### 2.3 RNA sequencing of human carotid plaques

RNA sequencing and analyses were conducted on carotid plaques from 82 patients, as described previously.^25^ The obtained gene expressions were presented as log2-transformed counts per million (CPM). Correlation between gene expression of *ASPN* and gene cell markers of monocytes (*CD14*), macrophages (*CD68, CD163, ITGAX*), endothelial cells (*CD34, VWF, PECAM1*), natural-killer cells (*NCAM1, B3GAT1*), mast cells (*KIT, FCER1A, FCER1G*), T cells (*CD3D, CD3E, CD4, CD8A, CD8B*), contractile SMCs (*ACTA2, TAGLN, MYH11*), synthetic SMCs (*KLF4, CD33*), fibroblast-like SMCs (*FN1, BGN, DCN, COL1A1*), and osteogenic-like SMCs (*RUNX2, MSX1, MSX2, ALPL*) were examined using Spearman correlation (rho).

Additionally, publicly available single-cell RNA sequencing data from carotid plaques was examined for the expression of ASPN across different cell types using the PlaqView explorer platform.^26^

### 2.4 Cell-type deconvolution of bulk RNA-seq data from human carotid plaques

Deconvolution of bulk RNA-seq data was performed using BayesPrism,^27^ a Bayesian model that jointly estimates cell-type proportions and cell-type-specific gene expression from bulk RNA-seq data, using single-cell reference data as prior information.

Briefly, single-cell reference data were obtained from an integrated single-cell analysis of human plaques,^28^ from which a subset comprising all carotid plaque cells (*n* = 113,146) was used. In total, 11 cell types (*B cells, dendritic cells, endothelial cells, fibroblasts, macrophages, mast cells, monocytes, natural killer cells, plasma cells, SMCs, and T cells)* were annotated among those carotid plaque cells. Gene markers highly expressed in each cell type were identified using the FindAllMarkers function from the Seurat package.^29^ To enhance cell-type specificity, minimize redundancy, and improve computational efficiency, up to the top 500 highly expressed marker genes per cell type were retained. Additionally, non-protein-coding, mitochondrial, ribosomal, and sex-chromosomal (X and Y) genes were removed to minimize potential batch effects and avoid sex-specific transcriptional biases. Outlier genes with expression >1% of total reads in >10% of bulk RNA-seq samples were further filtered out. Finally, the reference matrix, containing gene counts for a total of 2056 genes across 11 carotid plaque cell types, was used in deconvolution.

For a more granular cell type estimation, deconvolution was also performed using the same reference matrix with more detailed cell type annotations, i.e. B cell, dendritic cell (cDC1, cDC2), endothelial cell (EndoMT, intimal, lymphatic, proangiogenic), fibroblast, macrophage (foamy, inflammatory, resident), mast cell, monocyte, natural killer cell, plasma cell, SMC (fibromyocyte, SMC), and T cell (CD4^+^ effector, CD4^+^ regulatory, CD8^+^, CXCL8).

ASPN gene expression in SMCs was compared between symptomatic and asymptomatic patients. *IDO1* gene expression in SMCs and estimated cell type proportions were also compared between high- and low-ASPN plaques. *P*-values from Mann–Whitney *U* test were reported.

### 2.5 D-repeat polymorphisms

To call ASPN D-repeat polymorphisms from RNA-seq data, reads mapped to ASPN were extracted and converted to FASTQ format by SAMtools and BBTools.^30,31^ Next, a local *de novo* assembly by Trinity was conducted,^32^ followed by transcript quantification by salmon.^33^ D-repeat allele from the most abundant ASPN transcript per patient was then recorded.

### 2.6 Histo-/immunohisto-chemical stainings

Paraffin-embedded sections were used for ASPN immunohistochemistry, Alizarin Red S, and Von Kossa staining, whereas additional frozen sections were prepared for Oil Red O staining to visualize neutral lipids, and for Movat Pentachrome, CD68, glycophorin A, and cleaved collagen immunostaining.

#### 2.6.1 Histochemical analyses

Collagen was assessed using Movat Pentachrome staining, and neutral lipids were visualized with Oil Red O (Sigma-Aldrich, Darmstadt, Germany) staining.^30^ Plaque mineralization was evaluated using Alizarin Red S, which detects calcium ions, including amorphous forms, and Von Kossa, which specifically stains mineralized calcium phosphate but may miss amorphous calcium deposits or non-phosphate calcium salts.^31^ For the Alizarin Red S staining, tissue sections were deparaffinized, hydrated, and stained with a 0.0625% Alizarin Red S (Sigma-Aldrich) solution for 10 min in the dark, followed by phosphate-buffered saline (PBS) rinsing and counterstaining with 0.005% Fast Green (Sigma-Aldrich) until a pale green colour was observed under a microscope. Von Kossa staining involved immersion of sections in a 1% silver nitrate (Thermo Fisher Scientific Inc., Waltham, MA, USA) solution under ultraviolet light for 20 min, rinsing with distilled water, and incubation with 5% sodium thiosulphate (Thermo Fisher Scientific Inc.) for 5 min. Sections were then counterstained with 0.1% Nuclear Fast Red (Sigma-Aldrich) solution for 5 min, dehydrated, cleared in xylene, and mounted using Pertex. Plaques pre-incubated with EDTA (Thermo Fisher Scientific Inc.) served as negative controls for both mineralization stains.

#### 2.6.2 Immunohistochemistry

SMCs were identified using smooth muscle alpha-actin staining, while macrophages were detected with CD68 staining, as described previously.^30^ Intraplaque haemorrhage was assessed using Glycophorin A staining, as described previously.^30^ For collagen I and II cleavage detection, sections were probed using a C1,2C (Col 2 ¾ short, cleaved collagen) antibody, followed by biotinylated secondary antibody and DAB staining (Vector Laboratories, Inc., Newark, NJ, USA). For ASPN staining, sections were deparaffinized, rehydrated with xylene, ethanol and water, blocked with 5% bovine serum albumin (BSA), and incubated with primary antibody. A MACH3 probe and horseradish peroxidase polymer (Biocare Medical, Pacheco, CA, USA) were used to detect positive immunoreactivity. The slides were counterstained with Mayer’s Haematoxylin (HistoLab, Göteborg, Sweden) and dehydrated through graded alcohols to xylene before mounting. In immunohistochemical experiments, isotype controls were used to verify the specificity of primary antibody binding and eliminate non-specific background staining. A detailed description of antibodies used in immunohistochemistry is reported in Supplementary material online, *Table S3*.

The histological VI was calculated by (% CD68^+^ + glycophorinA^+^ + Oil Red O^+^ area)/(% smooth muscle α-actin^+^ + collagen^+^ area), as published previously.^30^

Slides were stained, scanned, and digitized using an Aperio ScanScope digital slide scanner (Aperio Technologies Inc., California, USA) and Olympus SlideView VS200 (Evident Scientific, Waltham, USA). Quantitative analyses were conducted on blinded samples utilizing BioPix iQ imaging software (version 2.3.1, Biopix AB). QuPath software was used for visualization and exporting images of stained sections. Results are presented as % positive area of the total plaque.

### 2.7 SMC culture

Immortalized SMCs from primary human coronary artery SMCs were generated as described previously.^32^ Cells were cultured in GlutaMAX DMEM Gibco™ (Fisher Scientific, Göteborg, Sweden) supplemented with 10% fetal bovine serum (FBS; Thermo Fisher Scientific Inc.), L-glutamine (Thermo Fisher Scientific Inc.), 100 U/mL penicillin and 100 μg/mL streptomycin (Thermo Fisher Scientific Inc.), and maintained at 37°C under 5% CO_2_. For ASPN overexpression, SMCs were transduced with either ASPN full-length sequence (Lenti ORF particles, Myc-DDK tagged) or control open reading frame (Lenti ORF control particles, pLenti-C-Myc-DDK) (OriGene Technologies, Inc., Rockville, MD, USA). Cell transduction was performed by incubating the target cells with retroviral supernatants at a multiplicity of infection of 10 for 24 h, followed by a 48 h rest period. Cells were expanded, and *ASPN* gene expression was monitored by quantitative real-time polymerase chain reaction (qRT-PCR) at every passage for a period of 4 weeks (see Supplementary material online, *Figure S5*).

### 2.8 Mineralization assay

SMCs were cultured for 12 days in normal or osteogenic medium, with medium refreshed every 2 days. Normal medium consisted of GlutaMAX DMEM (Gibco™) with 10% FBS, glucose (4.5 g/L), L-glutamine, 100 U/mL penicillin, and 100 μg/mL streptomycin. Osteogenic medium was additionally supplemented with 10 nM dexamethasone (Thermo Fisher Scientific Inc.), 10 mM β-glycerophosphate (Thermo Fisher Scientific Inc.), and 100 μM L-ascorbic acid 2-phosphate (Thermo Fisher Scientific Inc.). Cultures were fixed in 4% paraformaldehyde, stained with 2% Alizarin Red S for 30 min, and imaged via light microscopy. Staining was quantified by eluting dye with cetylpyridinium chloride (Thermo Fisher Scientific Inc.) and measuring absorbance at 570 nm using a Tecan Sunrise Microplate Reader (Tecan Trading AG, Männedorf, Switzerland).

### 2.9 Cell viability

Cell viability was assessed using the CyQUANT™ Direct Red Cell Proliferation Assay (Thermo Fisher Scientific Inc.), a cell-permeant DNA-binding fluorescent dye, following the manufacturer’s protocol. A masking dye was used to selectively block staining of dead cells or those with damaged membranes, allowing only healthy cells to be stained. Fluorescence intensity was measured in the CLARIOstar^®^ microplate reader (BMG LabTech, Ortenberg, Germany) and used to indicate cell viability across conditions.

### 2.10 qRT-PCR

SMC RNA was extracted with RNAeasy Plus Mini kit (Qiagen, Hilden, Germany) and cDNA was synthesized using High-Capacity RNA-to-CDNA™ kit (Applied Biosystems/Thermo Fisher Scientific Inc.). Gene expression was analysed by quantitative real-time PCR on QuantStudio 7 Flex instrument (Applied Biosystems/Thermo Fisher Scientific Inc.) using Taqman Fast Advanced master mix and appropriate Taqman probes (see Supplementary material online, *Table S2*). Relative mRNA levels were calculated using the 2^−ΔCt^ method, and glyceraldehyde-3-phosphate dehydrogenase (*GAPDH*) expression was used as endogenous control.

### 2.11 SMC lysates

SMCs were lysed with radioimmunoprecipitation assay (RIPA) buffer [150 mM NaCl (Thermo Fisher Scientific Inc.), 1.0% Triton X-100 (Sigma-Aldrich), 0.1% SDS (Sigma-Aldrich), 50 mM Tris (Sigma-Aldrich), pH 8.0] supplemented with a Thermo Scientific™ Halt™ Protease Inhibitor Cocktail (100X) (Thermo Fisher Scientific Inc.) to 1% final concentration. Protein concentrations were determined using the Pierce™ BCA Protein Assay Kit (Thermo Fisher Scientific Inc.), according to the manufacturer’s protocol.

### 2.12 Western blot

Proteins were separated by sodium dodecyl sulphate–polyacrylamide gel electrophoresis on Bio-Rad Mini-PROTEAN^®^ TGX Stain-Free™ gels (Bio-Rad Laboratories, Inc., California, USA) and transferred to polyvinylidene fluoride membranes using the Bio-Rad Trans-Blot Turbo System (Bio-Rad Laboratories, Inc., California, USA). The membranes were blocked with 5% BSA (Sigma-Aldrich) in Tris-buffered saline, pH 7.4 (Medicago AB, Uppsala, Sweden), containing 0.1% Tween-20 (Thermo Fisher Scientific Inc.), then incubated overnight at 4°C with primary antibodies (see Supplementary material online, *Table S3*). Immunodetection was performed with peroxidase-conjugated secondary antibodies and detected with chemiluminescent signals using Pierce™ Western Blot Signal Enhancer (Thermo Fisher Scientific Inc.). Bands were visualized by Bio-Rad ChemiDoc™ MP Imaging System and quantified using Image Lab Software (Bio-Rad Laboratories, Inc., California, USA).

### 2.13 Osteogenic ECM extraction

#### 2.13.1 Extraction

SMCs were seeded in 175 cm^2^ flasks and incubated in osteogenic medium for 12 days. The osteogenic ECM and cell monolayer were then washed twice with PBS and resuspended in matrix extraction buffer [PBS supplemented with 0.1% Triton X-100 (Sigma-Aldrich), 1 mM MgCl_2_ (Sigma-Aldrich), and 1 U/mL DNase I (Qiagen, Venlo, Netherlands)]. The samples were then incubated at 37°C for 2 h. Next, trypsin (T8802 from bovine pancreas; Sigma-Aldrich) was added to a final concentration of 0.001% (v/v), and the samples were incubated for an additional 15 h at 37°C.

#### 2.13.2 Purification

The isolated osteogenic ECM was washed twice with ice-cold dH_2_O, resuspended in dH_2_O, washed with chloroform and air-dried. The osteogenic ECM was solubilized in 2X NuPAGE lithium dodecyl sulphate sample buffer (Bio-Rad Laboratories, Inc., California, USA) with 2 M urea (Sigma-Aldrich) at 60°C.

#### 2.13.3 Protein separation

Proteins were separated on NuPAGE 4–12% Bis-Tris gels using 2-(*N*-morpholino)-ethanesulphonic acid (MES) running buffer and silver-stained using the Pierce™ Silver Stain for Mass Spectrometry (Thermo Fisher Scientific Inc.). Once the desired band intensity was reached, gel regions corresponding to 35–70 kDa were excised and destained.

#### 2.13.4 Mass spectrometry

The gel pieces from the above gels were subjected to in-gel digestion using a protocol adapted from Goodman *et al*. (2018).^33^ Pieces were destained in 50% ethanol in 50 mM HEPES pH 8.5 at 22°C with shaking at 650 rpm in a thermomixer for 15 min. This was repeated twice before dehydrating the gel in 100% ethanol at 22°C with shaking at 650 rpm for 5 min. The gel pieces were reduced and alkylated by incubating the pieces in 10 mM tris(2-carboxyethyl)phosphine and 40 mM chloroacetamide at 70°C for 5 min. Proteins were digested by covering the gel with trypsin (Promega) in 100 mM HEPES pH 8.5 at 10 ng/μL and incubating at 37°C overnight. The resulting supernatants containing released peptides were collected, and samples were desalted using a stage tipping protocol with C18 material (AttractSPE Disks Bio C18; Affinisep) as described previously^34,35^ but eluted with 0.1% trifluoroacetic acid in 50% acetonitrile. To allow detection of ASPN, analysis of peptide samples was performed on-line on a Dionex UltiMate 3000 (Thermo Fisher Scientific Inc.) coupled to a timsTOF Pro (Bruker Daltonics) mass spectrometer in the positive ion mode after separation using a C18 column at room temperature with gradient elution using 0.1% formic acid in water and 99.9% acetonitrile/0.1% formic acid. Data were obtained using either data-dependent acquisition (DDA) with parallel acquisition-serial fragmentation (DDA-PASEF) or data-independent acquisition parallel acquisition-serial fragmentation (DIA-PASEF) modes. DDA-acquired data were searched using FragPipe (v. 21.1) in MSfragger (v.4.0) against the human UniProt reference proteome (accession number UP000005640) including common contaminants with a false discovery rate (FDR) set at 1% (protein and peptide level), and both fixed and variable modifications. IonQuant (v. 1.10.12) was used for quantification with the match-between-run setting enabled and normalization disabled.^36–38^

### 2.14 Follow-up

Patients were followed up until December 2015 for cardiovascular events (CV), including post-operative myocardial infarction, stroke, transient ischaemic attack, *amaurosis fugax*, and cardiovascular death. The follow-up data were obtained from the Swedish National Board of Health and Welfare from the Swedish national inpatient register using International Classification of Diseases. The following ICD-10 codes were used to ascertain CV events: G45.3, G45.9, G46, I63.1-5, I63.8-9, and I64; I21-22, I24.8-9, I25.1-2, I25.5-6, I25.8.

### 2.15 Statistics

Shapiro–Wilk tests were utilized to assess the normality of the data. Variables following a normal distribution are presented as the mean with standard deviation (SD), whereas those showing non-normal distribution are given as median with interquartile range (IQR). For comparisons of continuous variables between two groups, the Mann–Whitney *U* test was employed. The Chi-squared test was used to compare categorical variables. Spearman's rank correlation was applied to assess associations between continuous variables. Adjustments for multiple comparisons were made, where appropriate, using the Benjamini–Hochberg method (FDR).^33^

Patients were stratified into high and low ASPN categories based on the median plaque ASPN levels. Kaplan–Meier estimator was used to analyse the association of plaque ASPN levels with post-operative cardiovascular events. The *P*-value for differences between groups was calculated using the log-rank test. Hazard ratios (HRs; with 95% confidence intervals) were obtained from a Cox proportional hazards regression, adjusted for age, sex, symptom, and diabetes. Statistical significance was determined at a *P*-value of <0.05. In the *in vitro* experiments, statistical analysis was performed using two-way ANOVA followed by Tukey’s post-hoc test. Statistical significance was indicated as follows: *P* < 0.05 (*), *P* < 0.01 (**), *P* < 0.001 (***), and *P* < 0.0001 (****).

Statistical analyses were conducted using RStudio (version 2024.04.0 + 735) and GraphPad Prism version 8.0.0 for MacOS (GraphPad Software, San Diego, CA, USA).

## 3. Results

### 3.1 Increased plaque ASPN in asymptomatic patients

**Figure 1:**
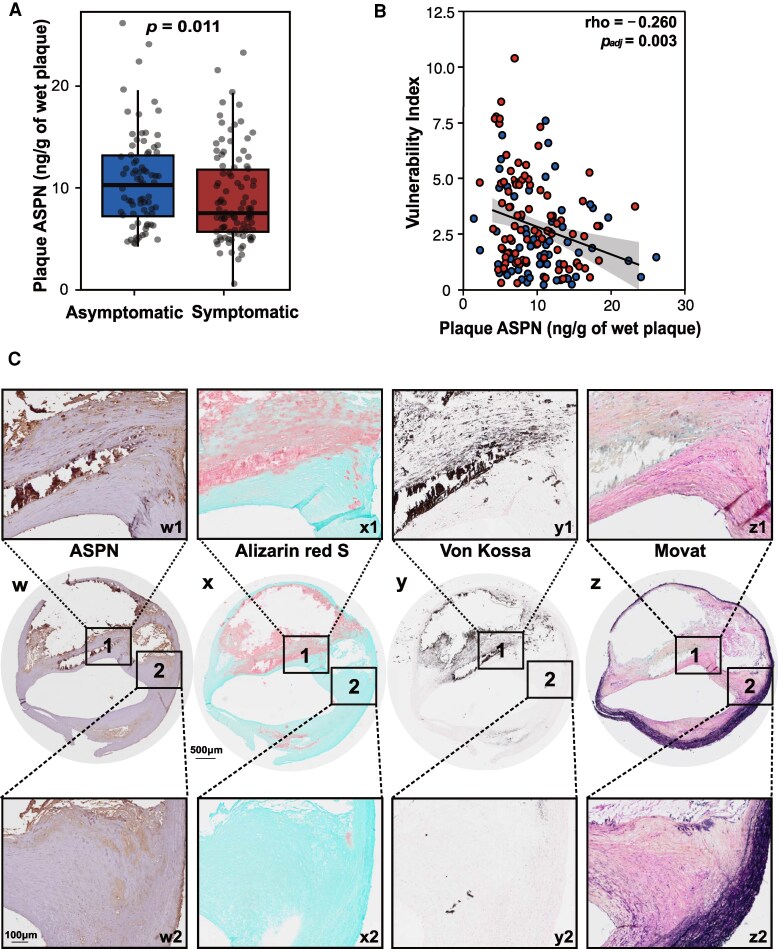
Elevated ASPN levels in asymptomatic patients and localization in calcified (1) and fibrotic regions (2). (*A*) ASPN protein levels (ng/g of wet plaque) were higher in plaques from asymptomatic patients (*n* = 78) compared with symptomatic (*n* = 98) (*P* = 0.011). The statistical significance was determined by Mann–Whitney *U* test. (*B*) Spearman correlation between vulnerability index and plaque ASPN (ng/g of wet plaque) in carotid plaques from symptomatic and asymptomatic patients (rho = −0.260; *p*_adj_ = 0.003). (*C*) Representative images from consecutive human carotid plaque slides immunostained for ASPN (w1, w2), or stained with Alizarin red (calcium deposits) (x1, x2), Von Kossa (calcium phosphate salts) (y1, y2) to visualize calcification, Movat pentachrome (z1, z2) to visualize collagen, elastin. Scale bars indicate 500 μm in the overview images and 100 μm in the corresponding magnified insets.

We analysed 176 carotid plaques from the CPIP cohort to investigate the association of ASPN expression in human atherosclerotic plaques and the plaque phenotype. The study participants were 67.6% male, the mean age was 70.1 years (SD, ±8.25) and the mean body mass index was 26.7 (SD, ±3.96). The median degree of stenosis was 90% (IQR, 80–95%), and 55.7% of the patients had suffered pre-operative cerebrovascular symptoms. Detailed baseline demographics and clinical laboratory data for the study cohort divided by high and low protein plaque ASPN levels are presented in *Table 1*. Baseline characteristics of the cohort stratified by symptomatic status and patient’s medication are provided in Supplementary material online, *Table S1*. Analysis of human carotid plaques with ELISA revealed that plaque ASPN levels were increased in the asymptomatic plaques [10.33 (IQR 7.25–13.16) ng/g of wet plaque] compared with symptomatic plaques [7.54 (IQR 5.71–11.80) ng/g of wet plaque, *P* = 0.011] (*Figure 1A*). ASPN D-repeat polymorphism was successfully called from a subgroup of 64 plaques from the bulk RNA-seq cohort (43 symptomatic, 21 asymptomatic). Among the identified ASPN D-repeat polymorphisms, the D14 allele was the most abundant, followed by D13 and D15 (see Supplementary material online, *Figure S2*). No significant differences in the frequencies of D-repeat alleles (D14, D13, and D15) were observed between symptomatic and asymptomatic patient plaques, suggesting that differences in ASPN expression between symptomatic and asymptomatic patients are less likely to be driven by ASPN D-repeat polymorphisms.

### 3.2 Plaque ASPN positively correlates with histological plaque stability features

Plaque protein levels of ASPN correlated positively with plaque area of SMC marker α-actin (rho = 0.193, *p*_adj_ = 0.025, *Table 2*). There were inverse correlations between plaque ASPN and plaque area of neutral lipids (Oil Red O; rho = −0.196, *p*_adj_ = 0.02) and with intraplaque haemorrhage (glycophorin A; rho = −0.259, *p*_adj_ = 0.002) (*Table 2*). Overall, plaque ASPN protein levels correlated inversely with the previously described^30^ plaque VI (rho = −0.260, *p*_adj_ = 0.003) (*Figure 1B*). In addition, the presence of ASPN in human plaque tissue was confirmed by immunohistochemistry (*Figure 1C*-*w*), and ASPN was observed in calcified areas visualized using Alizarin Red S (*Figure 1C*-*x*) and Von Kossa (*Figure 1C*-*y*) staining. ASPN was also observed in areas rich in collagen fibres (Movat staining) in proximity to the shoulder region (*Figure 1C*-*z*). Control stainings with isotype controls and EDTA-treated sections for ASPN and calcium staining, respectively, are provided in Supplementary material online, *Figure S3A*.

**Table 2:** ASPN plaque levels correlate with histological plaque vulnerability index and its components

Plaque features	rho	*P*	*P* adjusted^a^
*Histology (% area)*			
Vulnerability Index^a^	−0.260	0.001	0.003
Oil Red O	−0.196	0.01	0.020
Glycophorin A	−0.259	0.001	0.002
CD68	−0.076	0.340	0.446
α-Smooth muscle actin	0.193	0.014	0.025
Collagen	0.039	0.622	0.722

Spearman correlation coefficients (rho) are calculated to assess the relationships between ASPN plaque levels and a histological vulnerability index. The *P*-values are obtained from Spearman's rank correlation test. Adjusted *P*-values were calculated using the Benjamini–Hochberg method.

^a^Calculated as (% CD68^+^+glycophorin A^+^+Oil Red O^+^ area)/(% α-smooth muscle actin^+^+collagen^+^ area).

### 3.3 ASPN in plaques is associated with plaque stabilizing features and mineral deposition

When exploring how plaque ASPN protein levels were associated with plaque ECM components (*Table 3*), we observed that ASPN positively correlated with several plaque stabilizing features such as the TGF-β family (TGF-β2; rho = 0.561, *p*_adj_ < 0.001, TGF-β3; rho = 0.396, *p*_adj_ < 0.001), collagen (rho = 0.248, *p*_adj_ = 0.005) and elastin (rho = 0.423, *p*_adj_ < 0.001). In addition, plaque ASPN levels were significantly associated with multiple regulators of ECM turnover, such as matrix metalloproteinases (MMPs) and tissue inhibitors of metalloproteinases (TIMPs), while negatively correlated with histologically detected cleaved collagen (rho = −0.303, *p*_adj_ = 0.002). Specifically, ASPN showed positive correlations with MMPs (MMP3; rho = 0.175, *p*_adj_ = 0.058 and MMP10; rho = 0.221, *p*_adj_ = 0.014), as well as TIMPs (TIMP2; rho = 0.564, *p*_adj_ < 0.001 and TIMP3; rho = 0.305, *p*_adj_ = 0.024). Interestingly, plaque ASPN levels correlated negatively with the mineral deposition detected by Von Kossa histochemically (rho = −0.231, *p*_adj_ = 0.007).

**Table 3:** Plaque ASPN levels correlate with ECM components, calcium deposits, and matrix turnover components

Plaque matrix	rho	*P*	*P* adjusted^a^
***ECM components***			
TGF-β1 (pg/g of wet plaque)	0.175	0.033	0.062
TGF-β2 (pg/g of wet plaque)	0.561	1.27 × 10^−13^	1.62 × 10^−12^
TGF-β3 (pg/g of wet plaque)	0.396	6.14 × 10^−7^	2.85 × 10^−6^
Collagen (mg/g of wet plaque)	0.248	0.002	0.005
Elastin (mg/g of wet plaque)	0.423	5.6 × 10^−8^	3.1 × 10^−7^
***Calcium deposits***			
*Histology—*% positive area			
Von Kossa	−0.231	0.003	0.007
***Matrix turnover***			
*Matrix metalloproteinases (MMPs)*			
MMP2 (pg/g of wet plaque)	0.169	0.036	0.065
MMP3 (pg/g of wet plaque)	0.175	0.030	0.058
MMP9 (pg/g of wet plaque)	−0.148	0.070	0.106
MMP10 (pg/g of wet plaque)	0.221	0.006	0.014
*Tissue metalloproteinase inhibitors (TIMPs)*			
TIMP1 (pg/g of wet plaque)	0.087	0.289	0.377
TIMP2 (pg/g of wet plaque)	0.564	5.5 × 10^−14^	7.6 × 10^−13^
TIMP3 (pg/g of wet plaque)	0.305	0.011	0.024
*Histology—*% positive area			
Cleaved collagen	−0.303	7.3 × 10^−4^	0.002

Spearman correlation coefficients (rho) are presented to demonstrate the relationships between plaque ASPN with ECM plaque components and matrix turnover factors. The *P*-values are obtained using Spearman’s rank correlation test.

^a^Adjusted *P*-values were calculated using the Benjamini–Hochberg method.

### 3.4 *ASPN* levels correlate with SMCs populations in plaques

**Figure 2:**
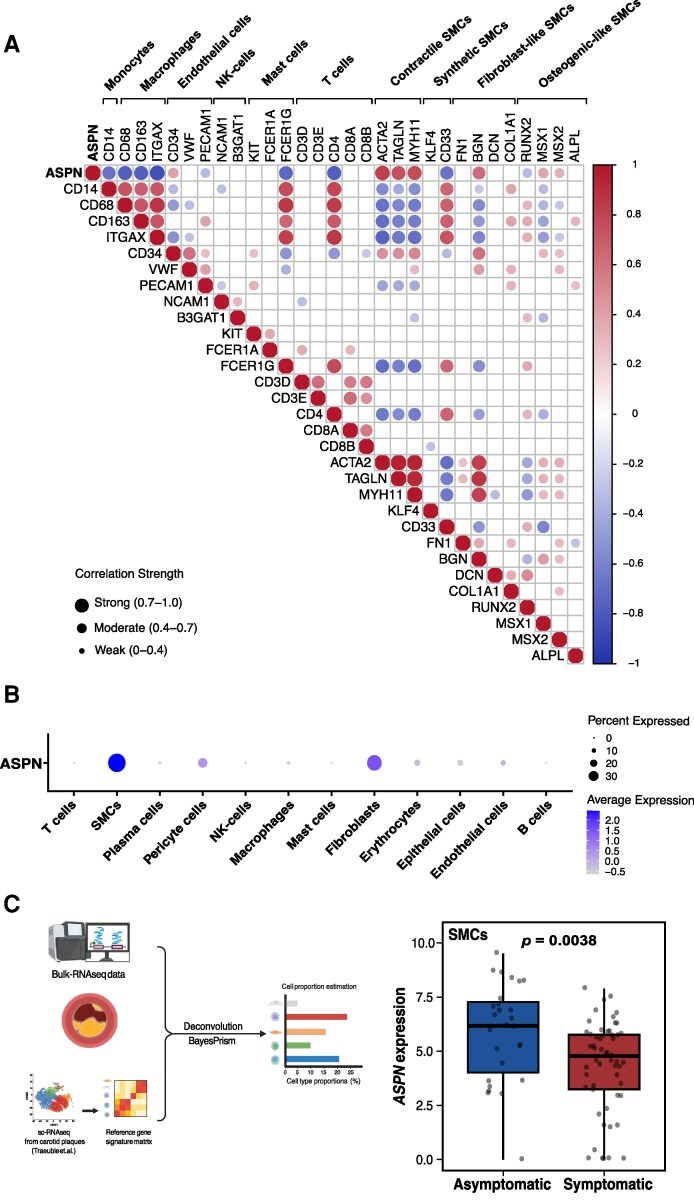
*ASPN* expression across vascular cell types in plaque bulk RNA and single-cell transcriptomic data. (*A*) Correlation heatmap of *ASPN* gene expression in different cell types. The heatmap displays Spearman correlation coefficients between log2CPM expressions of *ASPN* and various gene cell markers subtypes in carotid plaques from 82 patients. The genes analysed include markers for monocytes (*CD14*), macrophages (*CD68, CD163, ITGAX*), endothelial cells (*CD34, VWF, PECAM1*), natural-killer cells (*NCAM1, B3GAT1*), mast cells (*KIT, FCER1A, FCER1G*), T cells (*CD3D, CD3E, CD4, CD8A, CD8B*), contractile SMCs (*ACTA2, TAGLN, MYH11*), synthetic SMCs (*KLF4, CD33*), fibroblast-like SMCs (*FN1, BGN, DCN, COL1A1*), and osteogenic-like SMCs (*RUNX2, MSX1, MSX2, ALPL*). The colour gradient represents correlation coefficients ranging from −1 to 1, with blue indicating negative correlations, white indicating no correlation, and red indicating positive correlations. Only significant correlations (*P* < 0.05) are displayed. Gene names are shown along the *x* and *y* axes, and circle size indicates the strength of correlation: strong (0.7–1.0), moderate (0.4–0.7), and weak (0–0.4). (*B*) *ASPN* expression dot plot across different Seurat clusters using the PlaqView 2.0 platform.^26^ The dataset analysed included single-cell transcriptomic data from carotid artery plaques of 38 patients (5,633 cells).^39^ Each dot represents a specific cell cluster, with its size indicating the portion of cells within cluster expressing *ASPN.* Colour intensity reflects the average expression level. (*C*) Overview of the deconvolution workflow of bulk RNA-seq carotid plaque data. Box plot of ASPN expression [log(ASPN read counts + 1)] in relation to estimated SMC proportions in asymptomatic and symptomatic plaques. Statistical significance was assessed with the Mann–Whitney *U* test. Part of panel *C* created in BioRender. Fountas, P. (2025) https://BioRender.com/s8b55y9.

The mRNA levels of *ASPN* were assessed in a subgroup of 82 plaques using RNA sequencing. In line with the protein levels, *ASPN* gene expression levels were higher in asymptomatic plaques compared with symptomatic plaques [log_2_(fold change) = 1.23, *P* = 0.003; Supplementary material online, *Figure S3B*]. From the bulk RNA sequencing data analysis, we found that *ASPN* gene expression was negatively correlated with monocyte marker (*CD14;* rho = −0.670, *p*_adj_ < 0.001) and macrophage markers (*CD68;* rho = −0.765, *p*_adj_ < 0.001, *CD163;* rho = −0.761, *p*_adj_ < 0.001 and *ITGAX;* rho = −0.848, *p*_adj_ < 0.001, *Figure 2*). Negative correlations were observed with natural killer, mast and T cell markers. Of note, *ASPN* strongly positively correlated with contractile SMC markers (*ACTA2;* rho = 0.816, *p*_adj_ < 0.001, *TAGLN;* rho = 0.742, *p*_adj_ < 0.001 and *MYH11;* rho = 0.761, *p*_adj_ < 0.001), while a negative correlation was observed with synthetic SMCs (*CD33;* rho = −0.669, *p*_adj_ < 0.001). Interestingly, *ASPN* positively correlated with fibroblast-like SMC markers (*BGN;* rho = 0.626, *p*_adj_ < 0.001) and osteogenic-like SMC markers (*MSX1;* rho = 0.386, *p*_adj_ < 0.001 and *MSX2*; rho = 0.334, *p*_adj_ < 0.001), while negatively with runt-related transcription factor 2 (*RUNX2*); rho = −0.328, *p*_adj_ < 0.001 (*Figure 2A*). Furthermore, using publicly available single-cell RNA sequencing data^39^ assessed through the PlaqView platform, *ASPN* expression was predominantly observed in SMCs and fibroblasts clusters, in alignment with our bulk sequencing data (*Figure 2B*). Cell-type deconvolution of our bulk RNA-seq data demonstrated that ASPN expression in SMCs was significantly higher in plaques from asymptomatic than symptomatic patients (*Figure 2C*). Notably, gene set enrichment analysis (GSEA) revealed that high *ASPN* gene levels were enriched in the pathways related to vascular SMCs functions, ECM organization, collagen and calcium binding, as well as TGF-β signalling (see Supplementary material online, *Figure S4*). In a multivariable logistic regression model adjusted for α-smooth muscle actin-positive area, plaques associated with symptoms had significantly lower odds of high ASPN levels (OR ≈ 0.50, *P* = 0.033). α-Smooth muscle actin area was not a significant predictor for symptoms (*P* = 0.201), indicating that the association between ASPN and plaque phenotype is independent of SMC prevalence.

### 3.5 Low ASPN plaque levels predict an increased risk of post-operative cardiovascular events

**Figure 3:**
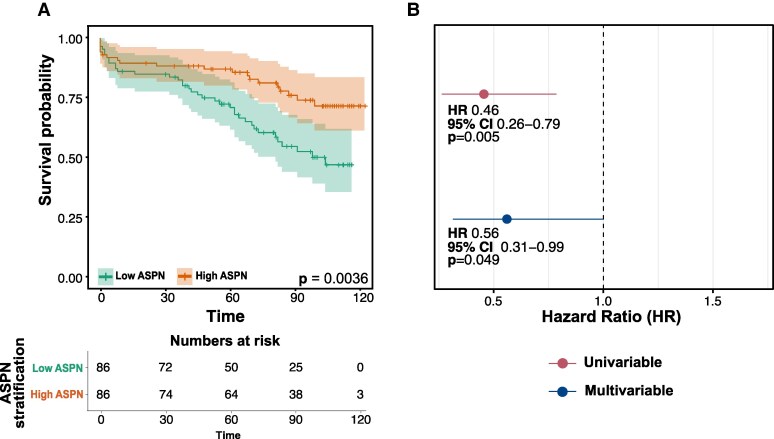
Inverse association between plaque ASPN protein levels and future cardiovascular (CV) events. (*A*) Kaplan–Meier curves for CV events were performed for ASPN levels by stratifying patients above (high ASPN, *n* = 86) and below the median (low ASPN, *n* = 86). Higher ASPN levels were associated with a lower risk of cardiovascular events (*P* = 0.0036 from log-rank test). (*B*) Univariate (HR of 0.46 (95% CI 0.26–0.79, *P* = 0.005) and multivariable (HR of 0.56 (95% CI 0.31–0.99, *P* = 0.049). Cox proportional hazard regression model adjusted for age, sex, symptom status, and diabetes for plaque ASPN in relation to CV events.

Next, we examined if plaque protein levels of ASPN were associated with future CV events. Interestingly, patients with higher ASPN protein levels [above median; median 12.52 (IQR 10.80–15.05) ng/g of wet plaque] had a lower risk of future CV events during a median follow-up period of 6.58 years (IQR 3.98–8.58) (*P* = 0.0036 from log-rank test) compared with those with lower levels of ASPN [below median; median 6.19 (IQR 4.97–7.25) ng/g of wet plaque] (*Figure 3A*).

A multivariable Cox regression model adjusted for well-known cardiovascular risk factors (age, sex, symptom status and diabetes), showed that above median ASPN protein levels were associated with a lower risk for future CV events, with a HR of 0.56 (95% CI 0.31–0.99, *P* = 0.049) compared with those with low intra-plaque ASPN protein levels (*Figure 3B*).

### 3.6 ASPN overexpression attenuates osteogenic calcification in vascular SMCs

**Figure 4:**
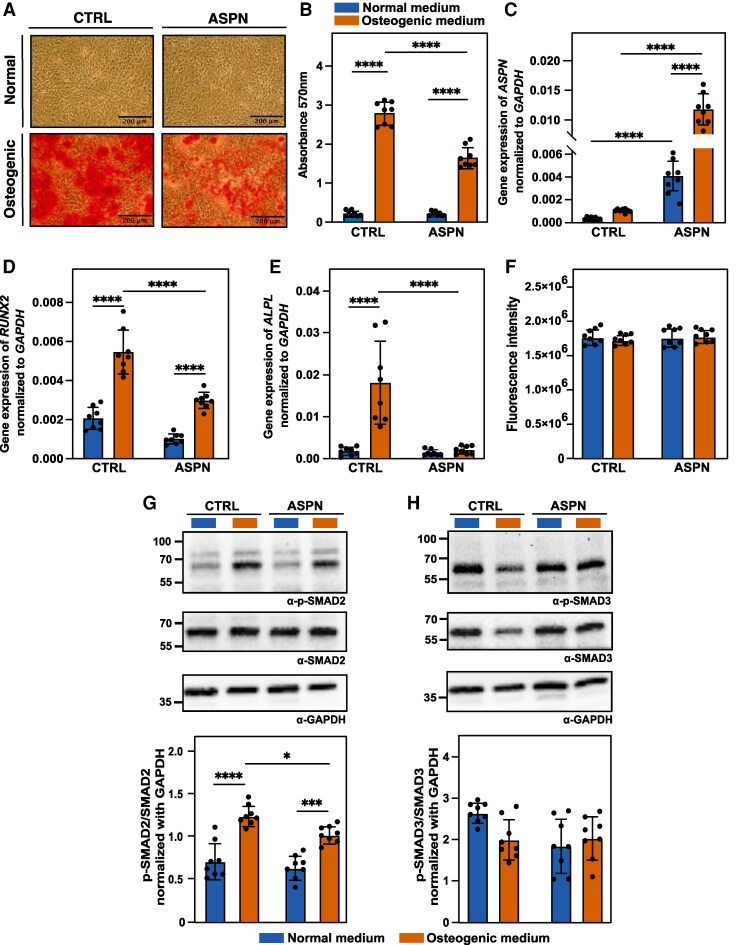
Effect of ASPN overexpression in calcification and osteogenic signalling in vascular SMCs. (*A*) Representative images of Alizarin Red S staining showing calcification in CTRL SMCs and ASPN overexpressing SMCs (ASPN SMCs) after 12 days in normal or osteogenic medium. Scale bar 200 μm is indicated. (*B*) Quantification of Alizarin Red S staining after elution with cetylpyridinium chloride and measuring absorbance at 570 nm. Calcification was reduced in ASPN SMCs compared with CTRL under osteogenic conditions. (*C*) Gene expression analysis of ASPN and osteogenic markers *RUNX2* and *ALPL* in SMCs (*D*, *E*) under normal and osteogenic conditions, normalized to *GAPDH*. Under osteogenic conditions, *RUNX2* and *ALPL* levels increased in both CTRL and ASPN SMCs but were significantly lower in ASPN compared with CTRL SMCs. (*F*) Cell viability assessment by CyQUANT™ DNA binding dye. The cell viability remained unchanged after the 12-day incubation. (*G*) Western blot analysis of phospho-SMAD2 (p-SMAD2) and total SMAD2. The p-SMAD2/total SMAD2 ratio was increased in both CTRL and ASPN overexpressing cells under osteogenic conditions but was lower in ASPN overexpressing cells. (*H*) Western blot analysis of p-SMAD3 and total SMAD3. No differences were observed in the p-SMAD3/total SMAD3 ratio. GAPDH was used as loading control. Values indicate ratios of the band pixel intensity of a phospho, and total protein normalized to GAPDH band pixel intensity. All experiments were repeated at least 8 times with bars indicating mean ± SD; the dots correspond to independent biological repeats for each condition. Statistical analysis was performed using two-way ANOVA followed by Tukey’s post-hoc test. Statistical significance was indicated as follows: *P* < 0.05 (*), *P* < 0.01 (**), *P* < 0.001 (***), and *P* < 0.0001 (****).

Given that ASPN was present in calcified regions in the plaque, associated with markers of osteogenic SMCs and correlated negatively with plaque calcification, we investigated whether ASPN could affect vascular mineralization processes. To achieve this, we overexpressed ASPN and assessed its impact on mineralization in an *in vitro* model using osteogenic medium treatment. A significant upregulation of *ASPN* gene expression was observed in ASPN overexpressing SMCs (ASPN SMC), and these elevated expression levels remained consistent across all cell passages used for experiments (see Supplementary material online, *Figure S5*). Treatment with osteogenic medium induced calcification in both control (CTRL) and ASPN SMCs compared with normal medium, as demonstrated by Alizarin Red S staining and quantification. However, ASPN SMCs exhibited significantly reduced calcification compared with CTRL SMCs (*P* < 0.0001; *Figure 4A* and *B*). Notably, *ASPN* gene expression was further increased in ASPN SMCs under osteogenic conditions (*P* < 0.0001), further emphasizing the heightened expression in response to the osteogenic medium (*Figure 4C*). Additionally, using a DIA-PASEF proteomics workflow, we detected 10 ASPN-derived peptides in ASPN SMCs osteogenic ECM vs. three present in CTRL SMCs osteogenic ECM (see Supplementary material online, *Figure S6*).

To assess the differentiation of SMCs, the gene expression of two osteogenic markers, *RUNX2* and alkaline phosphatase (*ALPL*), were measured. In CTRL cells, both *RUNX2* (*P* < 0.0001) and *ALPL* (*P* < 0.001) levels were significantly elevated under osteogenic conditions compared with normal medium. Importantly, ASPN overexpression resulted in significant lower expression of both markers compared with CTRL cells under osteogenic conditions (*RUNX2*, *P* < 0.001; *ALPL*, *P* < 0.01), suggesting an inhibitory effect on osteogenic differentiation (*Figure 4D* and *E*). There were no differences in SMC viability throughout the osteogenic differentiation conditions (*Figure 4F*).

Collagen is a primary scaffold for mineral deposition during osteogenic differentiation.^40^ In our *in vitro* system, while matrix collagen content increased under osteogenic conditions in both CTRL and ASPN overexpressing cells, ASPN overexpressing cells exhibited significantly lower collagen levels than CTRL cells under osteogenic conditions (see Supplementary material online, *Figure S7*).

Building on previous research indicating that TGF-β2 stimulation of SMCs upregulated collagen expression via SMAD2 signalling pathway,^41^ we examined the effect of ASPN overexpression on phosphorylation levels of SMAD2 at S255 residue (p-SMAD2) and SMAD3 at S423/S425 residue (p-SMAD3) under normal and osteogenic conditions. In osteogenic medium, the p-SMAD2/total-SMAD2 ratio increased in both CTRL (*P* < 0.0001) and ASPN SMCs (*P* < 0.001) compared with normal medium. However, ASPN overexpression resulted in a significant lower p-SMAD2/total SMAD2 ratio under osteogenic conditions (*P* < 0.05; *Figure 4G*), suggesting a potential dampening effect on SMAD2 activation. In contrast, the p-SMAD3/total SMAD3 ratio remained unchanged across all conditions (*Figure 4H*).

## 4. Discussion

In the present study, elevated carotid plaque ASPN levels were found in asymptomatic patients and were positively associated with features of plaque stability and ECM remodelling, while negatively associated with calcification. Longitudinally high plaque ASPN predicted lower numbers of cardiovascular events, and mechanistically, we showed its inhibitory role on matrix mineralization, in agreement with its possible role in plaque stabilization. Importantly, the inverse association between ASPN and symptomatic patient status remained significant after adjustment for SMC content, indicating that ASPN’s relationship with plaque stability is not solely attributable to its correlation with SMC abundance.

ASPN is implicated in several pathological diseases such as bone/joint disorders,^42^ cancer^43^ and cardiovascular conditions like heart failure^44^ and pulmonary arterial hypertension (PAH).^45^ ASPN in the lung tissue has a protective role in PAH,^45^ and during heart failure, ASPN protects against cardiac remodelling by reducing fibrosis and cardiomyocyte death.^46^

Plaques prone to rupture typically have lower levels of stabilizing ECM proteins,^20^ thinner protective caps, and increased degradation of collagen and other ECM proteins.^47^ These features are associated with an imbalance between ECM synthesis and degradation, as well as an altered activity of protease enzymes.^48^ Our findings indicate that high ASPN levels were associated with increased collagen, elastin, TGF-β2, and SMCs in plaques; this conclusion is supported by a previous proteomic study,^20^ which also reported elevated ASPN levels in stable ‘hard’ symptomatic collagen-rich plaques. These components are known to stabilize plaques and reduce rupture risk.^49^ Additionally, elevated ASPN levels were linked to decreased matrix turnover (positive correlation with TIMPs and inverse with cleaved collagen), further supporting a role of ASPN in plaque stability. Consistent with these results, high ASPN levels were associated with a reduced risk of cardiovascular events during follow-up.

One theory of vascular calcification involves elastin and collagen acting as scaffolds for calcium ion-binding through neutral sites, as proposed by Urry’s charge neutralization theory.^50^ This theory proposes that calcium binding induces localized positive charges on these proteins, attracting phosphate ions that neutralize the charges and facilitate crystal formation. The interplay between calcium, phosphate, and the ECM underlies the pathological mineralization observed in vascular tissues.^50^ Another theory proposes that plaque calcification is initiated by the phenotypic transdifferentiation of SMCs, through which they acquire osteoblast-like properties, further driving mineralization.^32^ In accordance with both theories, plaque ASPN localized in regions with different degrees of calcification, as well as collagen-rich regions, while an inverse correlation was documented with mineral deposition by Von Kossa staining.

Several studies suggest that calcified deposits depend on ECM composition.^51^ Given the proposed interaction of ASPN with fibrillar collagens, it is plausible that ASPN-mediated interactions contribute to the remodelling of collagen networks and calcium-binding dynamics during vascular calcification.

This proposal is supported by our observation that the ability of SMCs to mineralize ECM in response to osteogenic stimuli was reduced in the presence of elevated levels of ASPN. The differentiation of ASPN-overexpressing SMCs was significantly impaired, as evidenced by the markedly reduced expression levels of *RUNX2* and *ALPL*. This supports the notion that ASPN negatively modulates vascular calcification. In accordance, *ASPN* levels negatively correlated with *RUNX2* in our plaque bulk RNA sequencing data, further supporting the notion that ASPN reduces the transition of SMCs to an osteogenic-like phenotype.

Previous studies have demonstrated that ASPN inhibited chondrogenesis in articular cartilage in osteoarthritis by blocking the TGF-β/SMAD signalling pathway through direct interaction with all three TGF-β isoforms, dose-dependently suppressing SMAD2 phosphorylation.^52^ These findings align with our observation in plaques that the inhibitory effect of ASPN on calcification is mediated through the SMAD2 pathway. While our data indicate that ASPN promotes plaque stability via TGF-β-SMAD2 signalling and calcification, additional mechanisms may also be involved. ASPN activates the kynurenine pathway,^53^ which can modulate immune responses and CD8⁺ T cell survival. In our cell deconvolution of our bulk RNA-seq data, CD8⁺ T cell fractions were unchanged between high- and low-ASPN plaques (see Supplementary material online, *Figure S8A*), but SMC expression of IDO1—a key kynurenine pathway enzyme—was significantly increased in high-ASPN plaques (see Supplementary material online, *Figure S8B*). This suggests that ASPN may further stabilize plaques by enhancing IDO1-dependent inhibition of VSMC osteogenic differentiation,^54^ providing an additional possible mechanism to its effects on calcification.

This study focuses on samples from a cohort with advanced atherosclerosis, and the findings may not apply to the early stages of the disease. While supported by *in vitro* and follow-up data, the study cannot establish causality. Additionally, as the study examines only carotid plaques, the results may not extrapolate to other vascular beds, such as the coronary arteries.

In summary, the results of this study suggest that ASPN plays a role in vascular calcification associated with atherosclerosis, contributing to a plaque phenotype that is less prone to rupture. While additional research is necessary, these findings collectively point to a novel protective role of ASPN in atherosclerotic disease.

Translational perspective Vascular calcification contributes to plaque instability and subsequent clinical implications such as heart attacks and strokes. However, the biological factors that counteract vascular calcification in human atherosclerosis are not fully understood. In the present study, ASPN is associated with stable carotid plaques, lower histological plaque vulnerability, and reduced calcification. Functional experiments show that ASPN inhibits SMC osteogenic differentiation and matrix mineralization. Importantly, low intraplaque ASPN levels are linked to an increased risk of future cardiovascular events. Together, these findings suggest that ASPN could serve as a biomarker for plaque stability and as a therapeutic target to control vascular calcification and improve cardiovascular outcomes.

## Supplementary Material

cvag015_Supplementary_Data

## Data Availability

Due to the sensitive nature of the data collected in this study, as well as ethical and GDPR regulations, individual human data cannot be shared. However, an aggregated summary may be provided upon reasonable request to Prof Isabel Goncalves (isabel.goncalves@med.lu.se), subject to fulfilling adequate ethical and legal requirements in accordance with Lund University, Region Skåne and Swedish laws.
