# Supplementary material for: Elevated Asporin expression in human atherosclerotic plaques promotes their stability and reduces the risk for cardiovascular events
Source: Cardiovasc Res. 2026 Jan 20;122(3):349–62. doi: 10.1093/cvr/cvag015 (PMC13019687; doi:10.1093/cvr/cvag015)
Supplement: cvag015_Supplementary_Data [file cvag015_supplementary_data.zip › Figure S1.pdf]

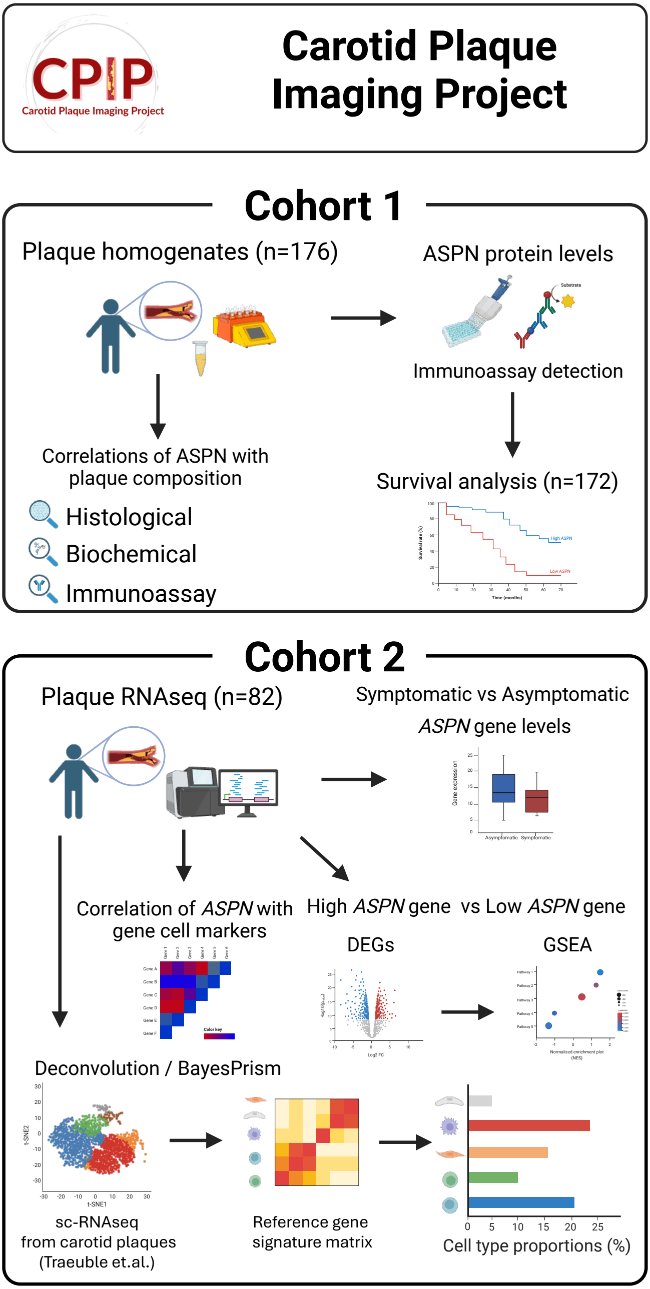
CPIP
Carotid Plaque Imaging Project
Carotid Plaque Imaging Project
Cohort 1
Plaque homogenates (n=176)
ASPN protein levels
Substrate
Immunoassay detection
Correlations of ASPN with plaque composition
Histological
Biochemical
Immunoassay
Survival analysis (n=172)
High ASPN
Low ASPN
Time (months)
Cohort 2
Plaque RNAseq (n=82)
Symptomatic vs Asymptomatic
ASPN gene levels
Gene expression
Asymptomatic
Symptomatic
Correlation of ASPN with gene cell markers
High ASPN gene vs Low ASPN gene
DEGs
GSEA
Gene A
Gene B
Gene C
Gene D
Gene E
Gene F
Color key
Log2 FC
Pathway 1
Pathway 2
Pathway 3
Pathway 4
Pathway 5
Normalized enrichment plot (NES)
Deconvolution / BayesPrism
t-SNE2
t-SNE1
sc-RNAseq from carotid plaques (Traeuble et.al.)
Reference gene signature matrix
Cell type proportions (%)
