# Supplementary material for: Elevated Asporin expression in human atherosclerotic plaques promotes their stability and reduces the risk for cardiovascular events
Source: Cardiovasc Res. 2026 Jan 20;122(3):349–62. doi: 10.1093/cvr/cvag015 (PMC13019687; doi:10.1093/cvr/cvag015)
Supplement: cvag015_Supplementary_Data [file cvag015_supplementary_data.zip › Figure S6.pdf]

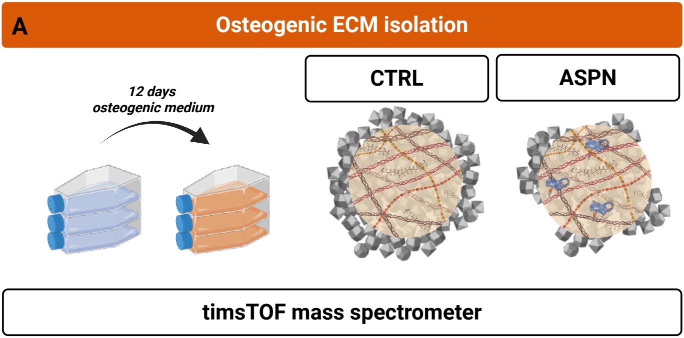

B

Peptide sequence	Osteogenic ECM: CTRL	Osteogenic ECM: ASPN
GLPPTLLELHLDYNK	not detected	detected
GLTSLYGLILNNNK	detected	detected
ISTVELEDFKR	not detected	detected
KIPSGLPELK	not detected	detected
LYLSHNQLSEIPLNLPK	detected	detected
RLYLSHNQLSEIPLNLPK	detected	detected
SLYSAISLFNNPVK	not detected	detected
VGVNDFCPTVPK	not detected	detected
VVHCSDLGLTSVPTNIPFDTR	not detected	detected
YWEMQPATFR	not detected	detected
