# Supplementary material for: Elevated Asporin expression in human atherosclerotic plaques promotes their stability and reduces the risk for cardiovascular events
Source: Cardiovasc Res. 2026 Jan 20;122(3):349–62. doi: 10.1093/cvr/cvag015 (PMC13019687; doi:10.1093/cvr/cvag015)
Supplement: cvag015_Supplementary_Data [file cvag015_supplementary_data.zip › Supplementary Material_figure_legends_.docx]

**Supplementary figures legends**

**Figure S1. Study design flow chart. Cohort 1**: Plaque homogenates (n = 176) were analyzed to quantify ASPN protein levels using immunoassay detection (ELISA). Correlations between ASPN protein levels and plaque components were assessed through histological, biochemical, and immunoassay techniques. The number of patients included in the correlations varied based on data availability for each specific variable. Survival analysis (n = 172) was performed to evaluate the association between ASPN protein levels and cardiovascular (CV) events. Four patients were excluded from the survival analysis due to bilateral carotid endarterectomy, with one side per patient removed to maintain data independence. **Cohort 2**: Plaque RNA sequencing (n = 82) was conducted to examine ASPN gene expression. The relationship between *ASPN* gene levels and cell-type-specific markers was explored. Differential expression gene (DEG) analysis and Gene Set Enrichment Analysis (GSEA) were performed to compare plaques with high versus low ASPN gene expression groups. Additionally, cell-type deconvolution of bulk RNA-seq data was performed with BayesPrism. Created in BioRender. Gialeli, C. (2025) <https://BioRender.com/s48u681>

**Figure S2. Distribution of ASPN D-repeat alleles in symptomatic and asymptomatic patients.** The plot displays the frequency distribution of ASPN D-repeat alleles in 64 patients (43 symptomatic, 21 asymptomatic). Blue bars indicate asymptomatic patients, and red bars indicate symptomatic patients. D-repeat alleles were called from RNA-seq data, with the allele from the most abundant transcript per patient used for analysis. Chi-squared tests were performed for each D-repeat allele to compare symptomatic versus asymptomatic groups.

**Figure S3. Control stainings and ASPN expression in symptomatic and asymptomatic patients.** **(A)** Control isotype staining for ASPN immunohistochemistry (**x1, x2**) and calcium histochemical stains with Alizarin red (**y1, y2**), Von Kossa (**z1, z2**) incubated with EDTA. **(B)** Plaque *ASPN* gene levels (log_2_CPM) in symptomatic and asymptomatic patients. log_2_[fold change] (log_2_FC) of *ASPN* expression compared to symptomatic patients was calculated by using the median values (log_2_FC=log_2_[median ASPN - asymptomatic] - log_2_[median ASPN - symptomatic], p=0.003).

**Figure S4. Functional associations of high plaque ASPN gene expression with SMC phenotype, ECM organization, collagen–calcium interactions, and TGF-β signaling.** The obtained bulk RNA sequencing data were stratified based on median *ASPN* gene expression levels. The identified differentially expressed genes (DEGs) between the two groups were then utilized to perform gene set enrichment analysis (GSEA) to identify significantly altered pathways using low *ASPN* group as reference. For the GSEA analysis, the curated gene sets Reactome/KEGG and Gene Ontology (GO) dataset were used as background. All analyses and visualization were performed using RStudio (version 2024.04.0+735). **(A)** The volcano plot shows the DEGs between the two groups, highlighting significantly up- and downregulated genes. **(B, C)** The ranked gene list for GSEA was created based on the signed -log10p_adj_ of the DEGs, using the clusterProfiler R package, incorporating both the direction and significance of the expression changes. The figures display dot plots for multiple pathways of interest. The x-axis shows the normalized enrichment score (NES), with customized gradient colors (blue to red) based on q-values and point sizes representing gene counts.

**Figure S5. *ASPN* expression monitoring every cell passage for a period of four weeks.** Gene expression analysis of *ASPN* on week 1, 2, 3 and 4 in control (CTRL) and ASPN overexpressing (ASPN SMCs), normalized to (*GAPDH*). All experiments were repeated at least 3 times; the dots correspond to independent biological repeats for CTRL and ASPN SMCs. Statistical analysis was performed using two-way ANOVA followed by Tukey’s post hoc test. Statistical significance was indicated as follows: p < 0.05 (*), p < 0.01 (**), p < 0.001 (***), and p < 0.0001 (****).

**Figure S6. ASPN detection by liquid chromatography-mass spectrometry (LC-MS/MS) in osteogenic ECM isolated by SMCs**. (**A**) Schematic representation of osteogenic ECM isolation from CTRL and ASPN smooth muscle cells (SMCs) culture, followed by LC-MS/MS analysis. (**B**) Table showing the presence or absence of ASPN peptides in CTRL and ASPN SMCs extracted osteogenic ECM. Created in BioRender. Gialeli, C. (2025) <https://BioRender.com/o45y170>

**Figure S7. ASPN overexpression affects collagen deposition under osteogenic conditions**. Cells were fixed with Kahle’s fixative (26% ethanol, 3.7% formaldehyde, 2% glacial acetic acid) for 15 minutes, washed with PBS, and stained with 0.1% Sirius Red (Direct Red 80 in 1% acetic acid) for 1 hour. After HCl washes, collagen-bound dye was eluted with NaOH. Absorbance was read at 540 nm (Tecan Sunrise Microplate Reader), and collagen content was quantified using a standard curve of rat tail collagen type I (Corning®). ECM collagen content in CTRL and ASPN SMCs under normal and osteogenic conditions. Under osteogenic conditions, ECM collagen content increased in CTRL and ASPN SMCs compared to normal medium. However, less collagen was produced by ASPN SMCs compared to CTRL upon osteogenic treatment. Collagen was quantified using a collagen standard curve and normalized to total lysate protein content. All experiments were repeated at least 8 times with bars indicating mean ± SD, the dots correspond to independent biological repeats for each condition. Statistical analysis was performed using two-way ANOVA followed by Tukey’s post hoc test. Statistical significance was indicated as follows: p < 0.05 (*), p < 0.01 (**), p < 0.001 (***), and p < 0.0001 (****).

**Figure S8. Association of CD8+ T cell proportions and SMC *IDO1* expression in relation to plaque *ASPN* levels. (A)** Box plot of CD8+ T cell proportions in patient plaques stratified into high and low *ASPN* expression groups based on median. **(B)** Box plot of *IDO1* expression (log[*IDO1* read counts + 1]) in the SMC fraction of plaques stratified by *ASPN* expression levels. Statistical significance was evaluated using the Mann–Whitney test.

**Figure S9. Unedited, full-length immunoblots corresponding to the cropped blots shown in the main Figure 4. (A)** Original blots for phospho-SMAD2 (p-SMAD2), total SMAD2 and GAPDH. The same membrane was first probed for p-SMAD2, then stripped and cut into sections to allow independent reprobing for total SMAD2 and GAPDH. The regions corresponding to the cropped blots shown in Figure 4G are indicated with red boxes. **(B)** Original blots for phospho-SMAD3 (p-SMAD3), total SMAD3 and GAPDH. The same membrane was first probed for p-SMAD3, then stripped and cut into sections to allow independent reprobing for total SMAD3 and GAPDH. The regions corresponding to the cropped blots shown in Figure 4H are indicated with red boxes. Exposure settings were applied uniformly across each full blot probed for each target.
