# Supplementary material for: Elevated Asporin expression in human atherosclerotic plaques promotes their stability and reduces the risk for cardiovascular events
Source: Cardiovasc Res. 2026 Jan 20;122(3):349–62. doi: 10.1093/cvr/cvag015 (PMC13019687; doi:10.1093/cvr/cvag015)
Supplement: cvag015_Supplementary_Data [file cvag015_supplementary_data.zip › Supplementary Material_tables.docx]

**Supplementary Tables**

**Table S1.** **Baseline clinical characteristics and medication in relation to symptoms status.** Mean with standard deviation (SD) for normally distributed continuous variables, median with interquartile range (IQR) for non-normally distributed continuous variables and frequencies (percentages) for categorical variables are presented. Characteristics of participants are compared using t-test for continuous normally distributed variables, Mann–Whitney U for continuous non-normally distributed variables and Pearson Chi-squared tests for categorical variables. HDL, high-density lipoproteins; LDL, low-density lipoproteins; hsCRP, high-sensitivity C-reactive protein.

|  | **Asymptomatic (n=78)** | **Symptomatic (n=98)** | **p** |
| --- | --- | --- | --- |
| Age, years (SD) | 67.26 (± 5.90) | 72.42 (± 9.12) | <0.001 |
| Sex: Men, n (%) | 51 (65.4%) | 68 (69.4%) | 0.688 |
| Degree of stenosis (IQR) | 90.00 (85.00 - 95.00) | 87.50 (75.00 - 95.00) | 0.055 |
| Diabetes, n (%) | 17 (21.8%) | 45 (45.9%) | 0.00153 |
| Hypertension, n (%) | 64 (82.1%) | 71 (72.4%) | 0.137 |
| Current smoking, n (%) | 34 (43.6%) | 22 (22.4%) | 0.005 |
| Body Mass Index (IQR) | 26.60 (23.63 - 29.30) | 26.15 (24.20 - 29.37) | 0.931 |
| hsCRP (mg/L) (IQR) | 2.00 (0.00 - 4.00) | 3.00 (2.00 - 6.25) | 0.00551 |
| Cholesterol (mmol/L) (IQR) | 4.20 (3.50 - 5.05) | 4.30 (3.68 - 5.10) | 0.573 |
| Triglycerides (mmol/L) (IQR) | 1.40 (0.95 - 1.80) | 1.30 (1.00 - 1.70) | 0.704 |
| HDL (mmol/L) (IQR) | 1.10 (0.88 - 1.43) | 1.06 (0.89 - 1.23) | 0.263 |
| LDL (mmol/L) (IQR) | 2.20 (1.80 - 3.02) | 2.55 (2.02 - 3.30) | 0.0997 |
| Statin treatment, n (%) | 72 (92.3%) | 84 (85.7%) | 0.258 |
| Anticoagulant treatment, n (%) | 77 (98.7%) | 91 (92.9%) | 0.136 |
| Betablockers treatment, n (%) | 35 (44.9%) | 45 (45.9%) | 1 |
| Antihypertensive treatment, n (%) | 65 (83.3%) | 80 (81.6%) | 0.924 |

**Table S2. Primers/probes used for RT-PCR (TaqMan^®^ Gene Expression Assays, Applied Biosystems)**

| **Gene** | **Primer sequence number** | **Base pairs** | |
| --- | --- | --- | --- |
| ASPN | Hs01550901_m1 | 158 |  |
| RUNX2 | Hs00899130_m1 | 102 |  |
| ALPL | Hs02758991_g1 | 93 |  |
| GAPDH | Hs02786624_g1 | 157 |  |

**Table S3. Primary and secondary antibodies used in Western Blotting (WB)**

| **Antibodies** | **Source** | **Host** | | **Dilution** | **Application** |
| --- | --- | --- | --- | --- | --- |
| ASPN | Abcam (ab201208) | Rabbit (Polyclonal) | 1:1000 | | IHC |
| C1,2C (Col 2 3/4Cshort) | IBEX Pharmaceuticals Inc. 50-1035 | Rabbit (Polyclonal) | 1:1000 | | IHC |
| p-SMAD2 (S255) | Abcam (ab188334) | Rabbit (Monoclonal) | 1:5000 | | WB |
| SMAD2 [EP784Y] | Abcam (ab33875) | Rabbit (Monoclonal) | 1:5000 | | WB |
| p-SMAD3 (S423+ S425) | Abcam (ab118825) | Rabbit (Polyclonal) | 1:5000 | | WB |
| SMAD3 [EP568Y] | Abcam (ab40854) | Rabbit (Monoclonal) | 1:5000 | | WB |
| GAPDH | Abcam (ab8245) | Mouse (Monoclonal) | 1:10000 | | WB |
| Goat anti-rabbit, Biotinylated IgG | Vector BA-1000 | Polyclonal | 1:2000 | | IHC |
| Rabbit IgG Isotype Control | Novus (NBP2-24891) | Polyclonal | 1:10000 | | IHC |
| Goat anti-rabbit IgG HRP | DAKO P0448 | Polyclonal | 1:5000 | | WB |
| Goat anti-mouse IgG HRP | DAKO P0447 | Polyclonal | 1:5000 | | WB |

*^*^All antibodies were diluted in 3% (w/v) BSA in TBS pH 7.4 with 0.1% Tween-20*
