# Supplementary figures and images for: Elevated Asporin expression in human atherosclerotic plaques promotes their stability and reduces the risk for cardiovascular events
Source: Cardiovasc Res. 2026 Jan 20;122(3):349–62. doi: 10.1093/cvr/cvag015 (PMC13019687; doi:10.1093/cvr/cvag015)

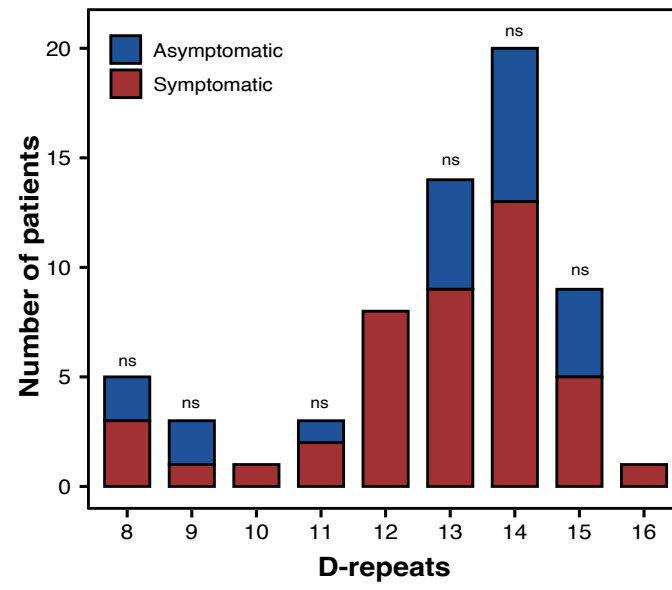

Supplement: cvag015_Supplementary_Data [file cvag015_supplementary_data.zip › Figure S2.pdf]

**A**

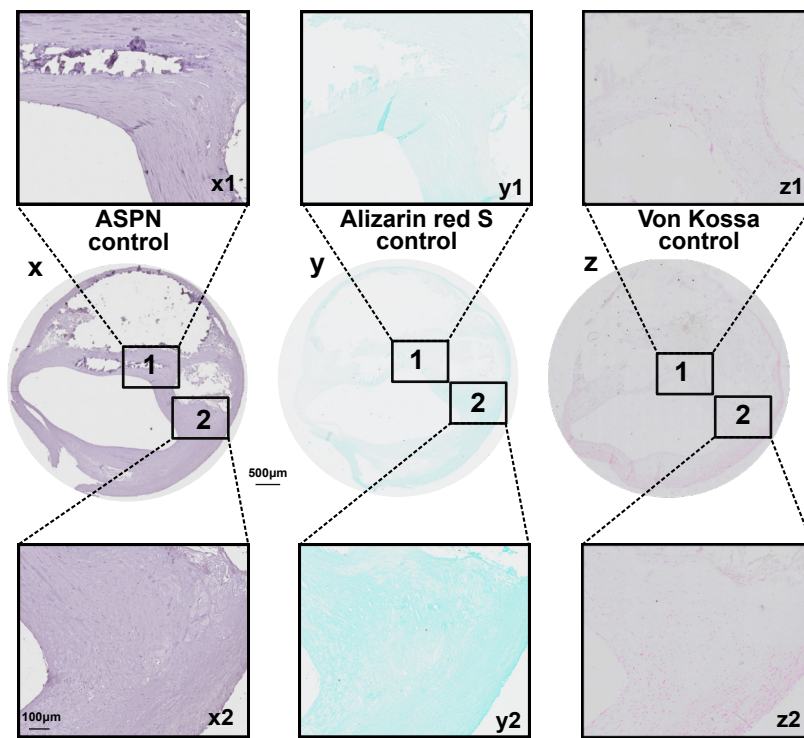

**B**

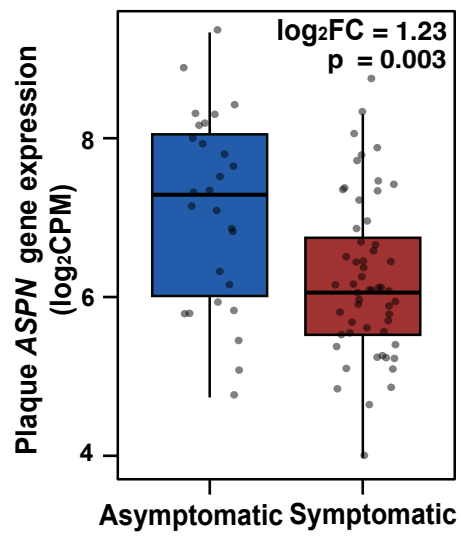

Supplement: cvag015_Supplementary_Data [file cvag015_supplementary_data.zip › Figure S3.pdf]

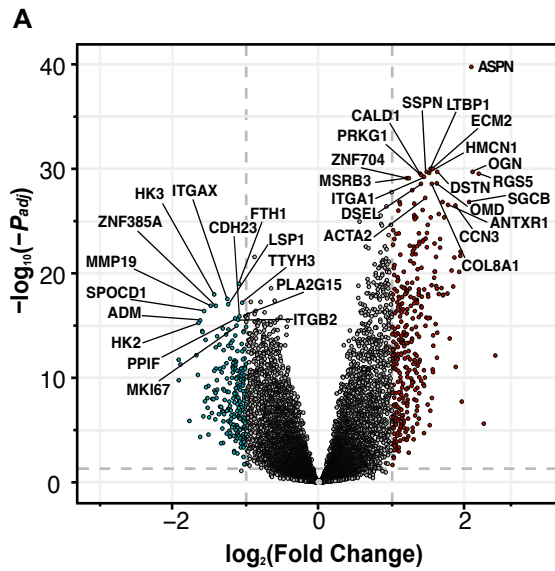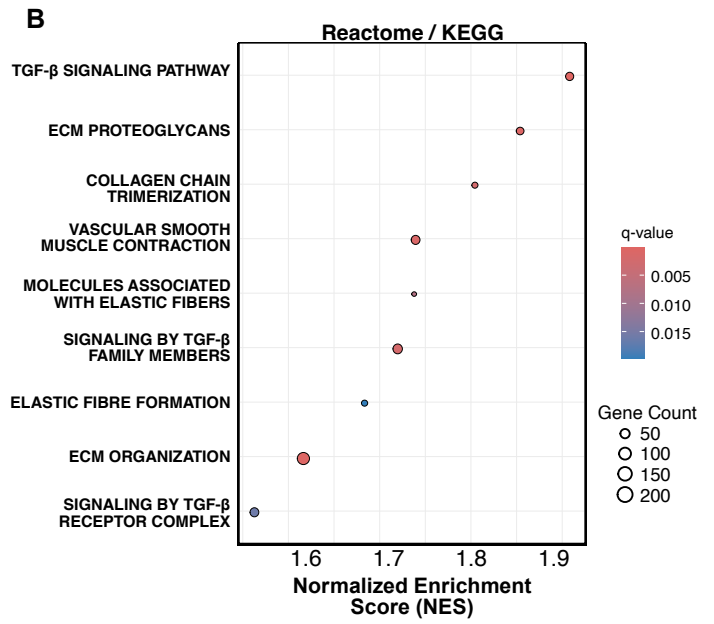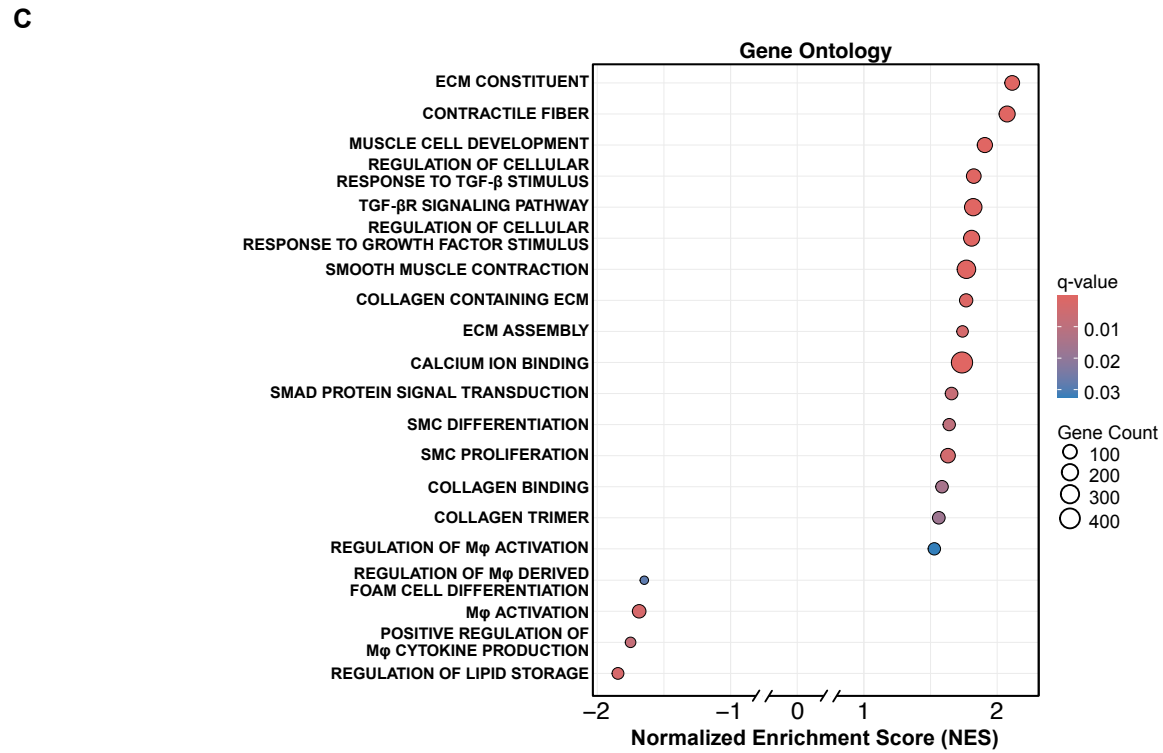

Supplement: cvag015_Supplementary_Data [file cvag015_supplementary_data.zip › Figure S4.pdf]

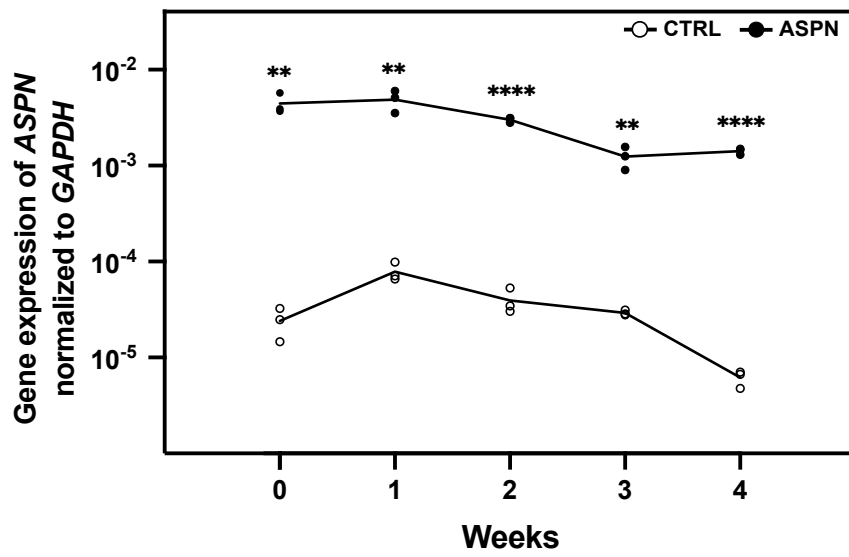

Supplement: cvag015_Supplementary_Data [file cvag015_supplementary_data.zip › Figure S5.pdf]

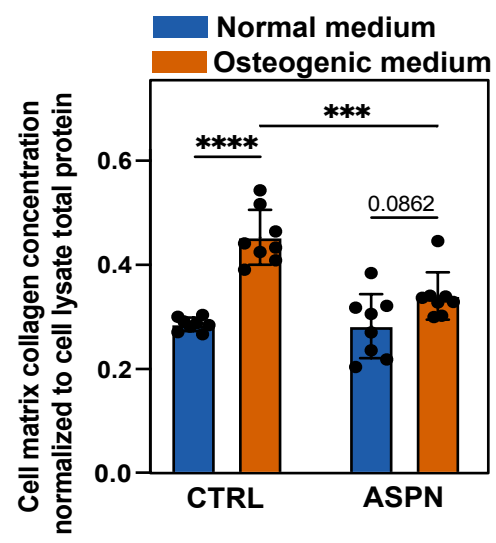

Supplement: cvag015_Supplementary_Data [file cvag015_supplementary_data.zip › Figure S7.pdf]

**A**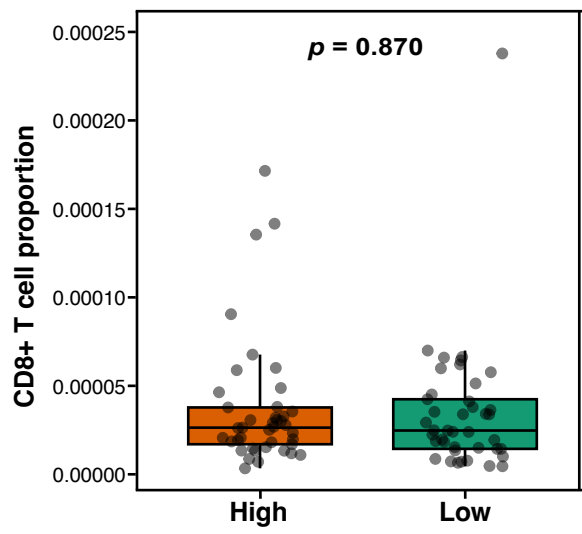**B**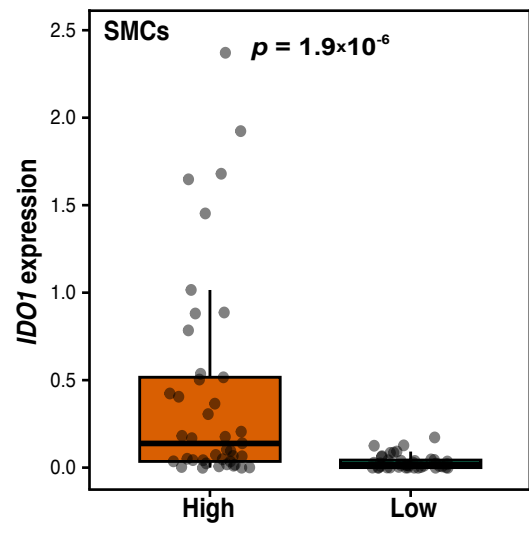

Supplement: cvag015_Supplementary_Data [file cvag015_supplementary_data.zip › Figure S8.pdf]

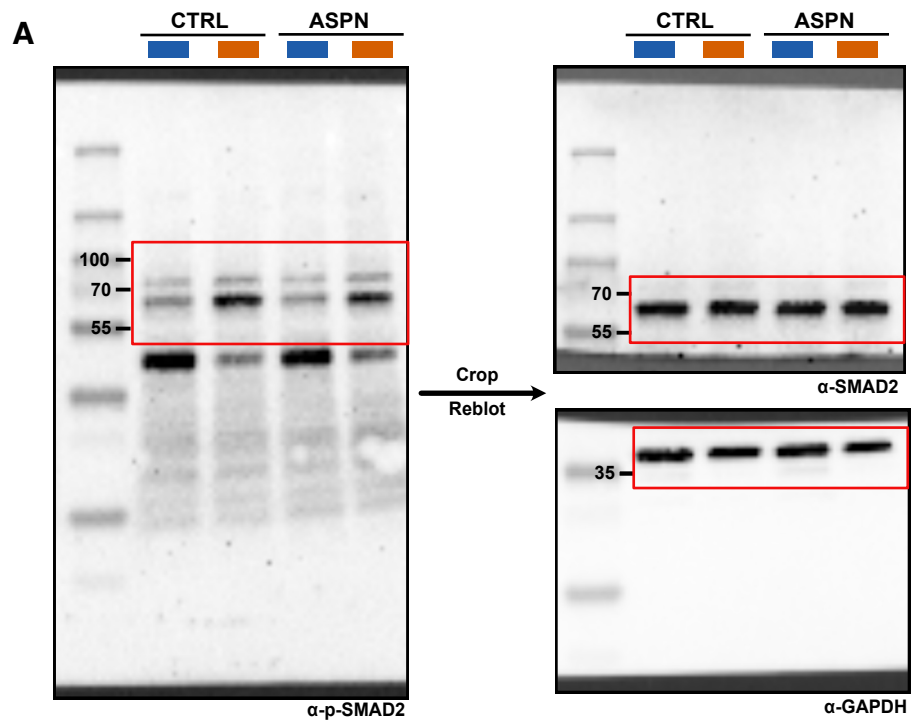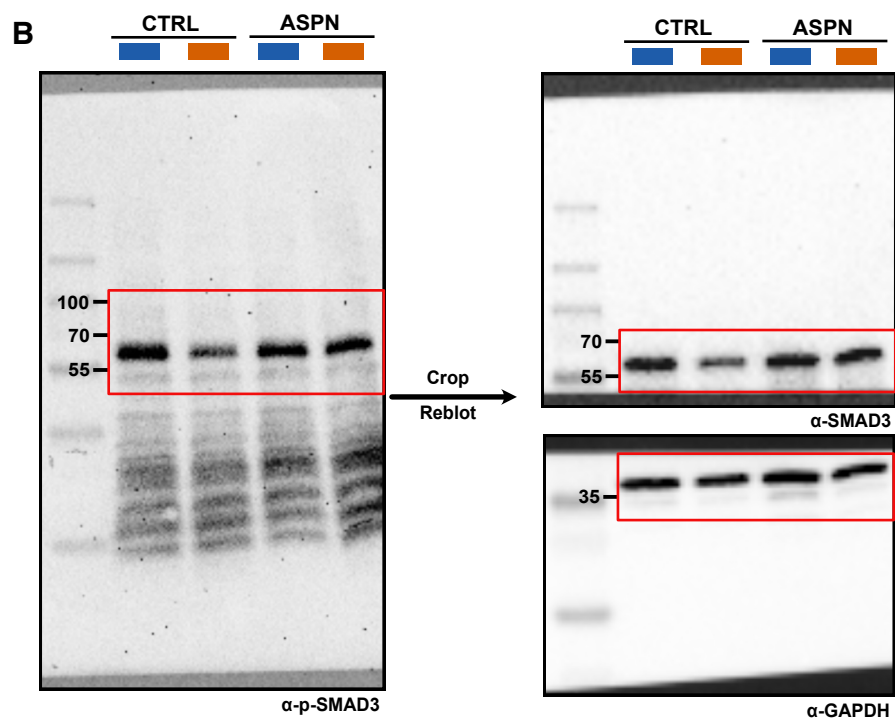

Supplement: cvag015_Supplementary_Data [file cvag015_supplementary_data.zip › Figure S9.pdf]
